# The Waiting Room Project: An Approach to Community Health Education in Hepatitis B

**DOI:** 10.4269/ajtmh.20-0232

**Published:** 2020-07-08

**Authors:** Nasreen S. Quadri, Jose D. Debes

**Affiliations:** Departments of Internal Medicine and Pediatrics; University of Minnesota; Minneapolis, Minnesota; E-mail: quadr015@umn.edu; Department of Medicine; Division of Infectious Diseases and International Medicine,; Division of Gastroenterology and Hepatology; University of Minnesota; Minneapolis, Minnesota; E-mail: debes003@umn.edu

Dear Sir,

The global burden of viral hepatitis, particularly hepatitis B, has continued to increase between 1990 and 2013, encompassing deaths, years lived with disability, and resultant disability-adjusted life years, thus impacting on both quality and quantity of life.^1^ Resource-limited areas, such as many areas in sub-Saharan Africa, suffer a disproportional brunt of this burden. Of concern is the general lack of awareness about hepatitis B and its silent progression among the communities living in high hepatitis B–endemic areas. Indeed, a previous study carried out by our group in Tanzania revealed low hepatitis B vaccine awareness and knowledge of personal serostatus among healthcare workers in a country where prevalence of hepatitis B surface antigen in the blood is > 8%.^2,3^

In the March 2020 issue of the *Journal*, Boye et al.^4^ reported that nearly two-thirds of a rural population interviewed in Senegal recognized the sequelae of chronic hepatitis B virus infection, although only one-third reported familiarity with the terminology “hepatitis B” as used by medical personnel. Their qualitative study revealed a disconnect between community perception of hepatitis B disease manifestations and the medical terminology used by healthcare workers in communicating about hepatitis B. We applaud the authors for their effort to identify cultural barriers in health care. We agree with them in their conclusion that eliciting known information from the community with specific attention to language surrounding hepatitis B is a significant first step in preparing to communicate biomedical information and dispel misinformation.

One approach to engage the community in health education in sub-Saharan Africa is what we have coined “The Waiting Room Project.” Patients in sub-Saharan Africa frequently spend more time waiting to be seen for medical care than participating in their medical consultation. This is a direct result of both healthcare worker shortages and inadequate infrastructure to coordinate scheduled appointments.^5^ We opted to use patients’ time in waiting rooms as an opportunity for small group education on health topics. We are currently using this model for health education on hepatitis B in Tanzania. A healthcare worker with training on how to teach about hepatitis B introduces the topic to a group of patients in the waiting room, aiming to provide important information in a 5- to 10-minute period, to maximize attention span. A survey of knowledge about hepatitis B is administered before the teaching session. Patients’ perceptions and curiosities are elicited through the process, and the healthcare worker is able to tailor education and engage with community members by answering questions important to them. The approach to this project is scalable by training healthcare workers, particularly in remote settings, to provide health education focused on disease prevalence, availability of testing, and prevention techniques. We believe that this approach can increase awareness, overcome cultural barriers, and open a potential avenue to achieving the WHO hepatitis B elimination target of 2030.^6^
